# Stepwise Release of Biologically Active HMGB1 during HSV-2 Infection

**DOI:** 10.1371/journal.pone.0016145

**Published:** 2011-01-19

**Authors:** Chloé Borde, Stéphanie Barnay-Verdier, Claire Gaillard, Hakim Hocini, Vincent Maréchal, Joël Gozlan

**Affiliations:** 1 Centre de Recherche des Cordeliers, Université Pierre et Marie Curie, Université Paris Descartes, INSERM, UMRS872, Paris, France; 2 Institut Mondor de recherche biomédicale, INSERM U955, Hôpital Henri Mondor, Créteil, France; 3 Service de Bactériologie-Virologie, Hôpital Saint-Antoine, Paris, France; Johns Hopkins University - Bloomberg School of Public Health, United States

## Abstract

**Background:**

High mobility group box 1 protein (HMGB1) is a major endogenous danger signal that triggers inflammation and immunity during septic and aseptic stresses. HMGB1 recently emerged as a key soluble factor in the pathogenesis of various infectious diseases, but nothing is known of its behaviour during herpesvirus infection. We therefore investigated the dynamics and biological effects of HMGB1 during HSV-2 infection of epithelial HEC-1 cells.

**Methodology/Principal Findings:**

Despite a transcriptional shutdown of HMGB1 gene expression during infection, the intracellular pool of HMGB1 protein remained unaffected, indicating its remarkable stability. However, the dynamics of HMGB1 was deeply modified in infected cells. Whereas viral multiplication was concomitant with apoptosis and HMGB1 retention on chromatin, a subsequent release of HMGB1 was observed in response to HSV-2 mediated necrosis. Importantly, extracellular HMGB1 was biologically active. Indeed, HMGB1-containing supernatants from HSV-2 infected cells induced the migration of fibroblasts from murine or human origin, and reactivated HIV-1 from latently infected T lymphocytes. These effects were specifically linked to HMGB1 since they were blocked by glycyrrhizin or by a neutralizing anti-HMGB1 antibody, and were mediated through TLR2 and the receptor for Advanced Glycation End-products (RAGE). Finally, we show that genital HSV-2 active infections also promote HMGB1 release in vivo, strengthening the clinical relevance of our experimental data.

**Conclusions:**

These observations target HMGB1 as an important actor during HSV-2 genital infection, notably in the setting of HSV-HIV co-infection.

## Introduction

High mobility group box 1 (HMGB1), an abundant nuclear protein, is the main prototype of the “alarmins”, a group of molecules that contribute to establishing immunity in response to cell damage. Extracellular HMGB1 derives either from active secretion by immunocompetent cells or from release by necrotic cells and by some apoptotic cells, a process that may be regulated at least in part by autophagy [1], [2]. Once outside the cell, HMGB1 coordinates various cellular responses, linking septic or aseptic stress signals to innate immunity and tissue repair. Importantly, HMGB1 extracellular activity is modulated by post-translational modifications. Notably oxidation of HMGB1 has been regarded as an important mechanism to negatively or positively regulates its extracellular activities [3], [4], [5]. The first HMGB1 receptor to be identified was RAGE [6], but HMGB1 also contributes to the activation of several immune receptors, including TLR-2 and -4 [7]. Extracellular HMGB1 can act by itself and/or in association with molecules such as CpG DNA, LPS and IL-1β [8].

Whereas the role of HMGB1 during bacterial infections has been extensively investigated, notably during severe sepsis [9], its dynamics and potential impact during viral infections remain largely unknown. In particular, the possible contribution of HMGB1 to the signalling or modulation of HSV-2 infection has not yet been addressed.

The prevalence of HSV-2 infection, the main cause of genital herpes, is growing. It reaches 30% among pregnant women in western countries and is even higher in selected populations and developing countries. Importantly, between 60% and 95% of HIV-infected individuals are also infected by HSV-2 [10]. Observational and experimental studies have shown a deleterious effect of HSV-2 on both HIV-1 transmission and disease progression [11]. Recent proof-of-concept trials have examined the impact of anti-herpetic therapy on HIV-1 viral load in plasma and/or genital secretions [12] [13] [14].

A growing set of arguments suggests that HMGB1 could play a significant role during HSV-2 infection. First, both epithelial cell damage and immune activation are observed during HSV-2 infection, and these two events may promote local HMGB1 release. Furthermore, soluble factors present in the genital tract, including CXC- and CC-type chemokines and interferon-β are crucial for an efficient immune response to HSV-2 [15]. The release of these molecules is triggered at least in part by interaction of cellular and/or viral components with several TLRs, particularly TLR-9 and TLR-2 [16]. TLR-2 is a known receptor for HMGB1, and HMGB1 has been shown to stimulate TLR-9 activation by DNA species in a RAGE-dependent manner [17]. Finally, some forms of HMGB1 act as chemoattractants or pro-inflammatory cytokines, and may modulate HIV-1 expression. These activities could be critical during HSV-2 infection, especially in a context of HIV-1 co-infection.

However HMGB1 dynamics and biological activities during active herpes simplex infection are especially difficult to predict. First, some HSV-2 gene products, such as ICP-10, can either be pro- or anti-apoptotic according to the cell type [18]. In the context of infection, the process of HSV-induced cell death, results from an even more complex balance between pro- and anti-apoptotic signals, which is also influenced by both the cell type and the origin of the viral strain [19], [20]. In addition, since numerous biologically active factors are released during HSV-2 infection, the precise contribution of HMGB1 needs to be determined.

This study was designed to examine three important issues. We first analyzed the modulation of HMGB1 transcription and dynamics during HSV-2 infection of epithelial cells from endometrial origin, with respect to virus multiplication and virus-induced cell damage. We then examined whether HMGB1 released during HSV-2 infection is biologically active. Finally, we studied HMGB1 accumulation in vivo in the genital tract of HSV-2-infected women.

## Results

### HSV-2 infection inhibits HMGB1 transcription in HEC-1 cells

HEC-1 cells, derived from a human endometrial cancer, were used as a model of epithelial cells. As HSV infection can repress the expression of cellular proteins through transcriptional and post-transcriptional mechanisms [21], [22], we examined whether HSV-2 infection down-regulates HMGB1 expression as well.

**Figure 1:**
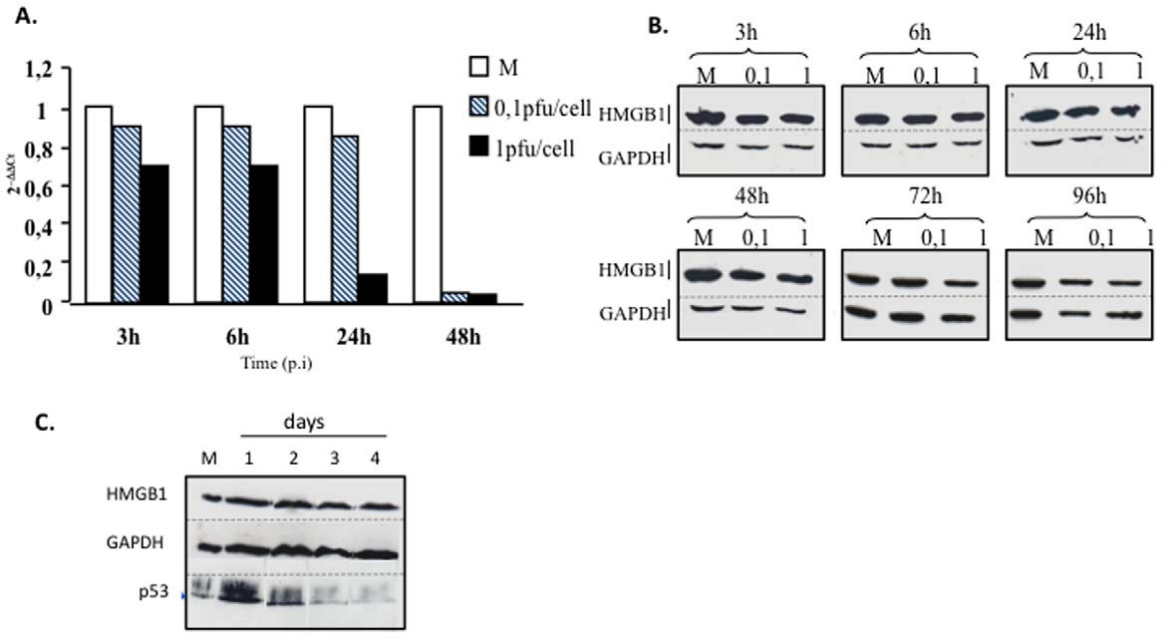
HMGB1 transcription and expression during HSV-2 infection. HEC-1 cells were infected at 0.1 or 1.0 pfu/cell. A time-course analysis of HMGB1 mRNA (A) and protein (B) was performed by quantitative RT-PCR and western blot, respectively, with 18S rRNA and GAPDH protein as controls. RNA quantification was performed according to the 2^ - ΔΔCT^ method [50]. (C) HMGB1 was detected more than 4 days in HEC-1 cells treated with actinomycin D. GADPH and P53 were used as controls. M  =  mock-infected cells. Results are representative of 3 independent experiments.

A marked decrease in HMGB1 transcription was observed as early as 3 h post-infection (pi) and reached 98% after 48 h (Figure 1A). Surprisingly, the HMGB1 protein level was barely affected (Figure 1B). This remarkable stability of HMGB1 was not directly due to HSV-2 infection, as it was also observed in uninfected cells treated with actinomycin D (Figure 1C).

### HSV-2 replicates and induces both apoptosis and necrosis in HEC-1 cells

**Figure 2:**
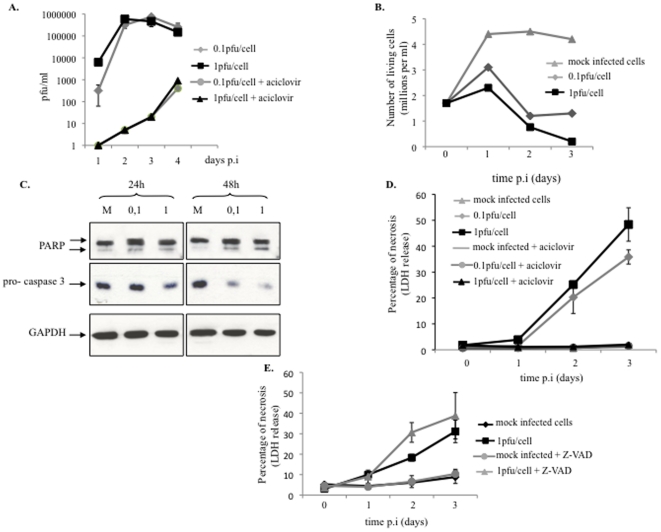
HEC-1 cell death during HSV-2 infection. HEC-1 cells, infected at 0.1 or 1.0 pfu/cell, were analyzed at various times post-infection for (A) HSV-2 productive infection, assessed by plaque assay, (B) cell viability, assessed by trypan blue exclusion, (C) apoptosis, assessed by western blot against PARP and pro-caspase 3, and (D) necrosis, assessed by extracellular LDH release over total LDH. (E) Necrosis was measured in mock and HSV-2-infected cells in the presence and absence of Z-VAD, an inhibitor of apoptosis. Results are either representative of at least 3 independent experiments (A, B, C) or are means and SD from 3 independent experiments (D, E).

Virus-associated cell damages were analyzed in relation with virus multiplication. A strong cytopathic effect was observed in most of the cells infected at 1 pfu/cell at day 1 pi (not shown). Virus production reached a plateau at day 2 pi for the two MOIs used (Figure 2A). Cell proliferation was inhibited on day 1, and cell viability was reduced on days 2 and 3 (Figure 2B). Virus-associated apoptosis, assessed by measuring PARP cleavage products and pro-caspase 3 expression, started at 24h and peaked at 48 h pi (Figure 2C), when viral production already reached a plateau (Figure 2A). Marked chromatin condensation was also observed microscopically in about 20% of cells on day 2 pi (not shown). Necrosis occurred later, as shown by LDH release, which was apparent on day 2 pi and increased over time in a dose-dependent manner (Figure 2D). Importantly, necrosis required viral replication, as it was prevented by aciclovir.

As both apoptosis and necrosis occurred sequentially, we intended to evaluate the respective contribution of primary and post-apoptotic necrosis. Inhibition of virus-mediated apoptosis by the peptide Z-VAD did not decrease the necrotic process, suggesting that necrosis did not occur as a consequence of apoptosis. Rather treatment with Z-VAD increased necrosis at day 2 (Figure 2E), showing that virus-induced apoptosis prevent necrosis in a significant number of HSV-2-infected cells.

### HMGB1 mobility during HEC-1 cell infection by HSV-2

**Figure 3:**
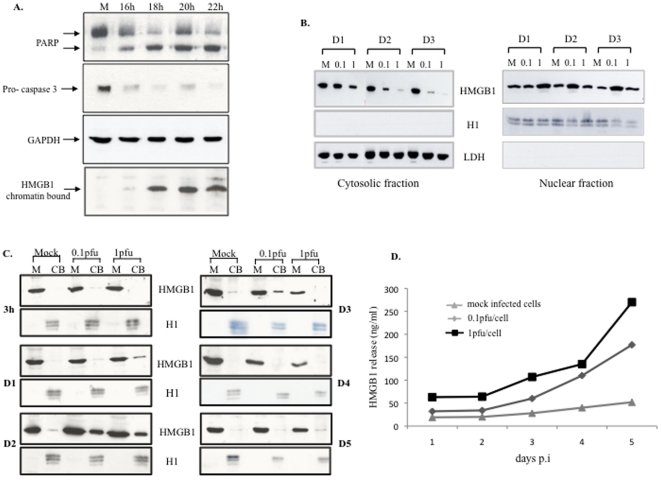
HMGB1 mobility is altered by HSV-2 infection. HEC-1 cells were treated with TNF-α (2 ng/ml) and CHX (10 µg/ml). At various times post-induction, western blot was performed on whole cell extracts to detect PARP and pro-caspase 3. A nuclear fraction obtained after NP-40-induced membrane permeabilization was also subjected to western blot to detect chromatin-bound HMGB1 (bottom). (A) Time-course analysis of HMGB1 in the cytoplasm and nucleus after HSV-2 infection of HEC-1 cells at 0.1 and 1.0 pfu/cell. M  =  mock-infected cells. (B) Time-course analysis of mobile (M) and chromatin-bound (CB) HMGB1 during HSV-2 infection, following membrane permeabilization by NP40. (C) Measurement of extracellular HMGB1 by ELISA after infection of HEC-1 cells by HSV-2. Results are representative of 3 independent experiments.

HMGB1 binds tightly to chromatin during some forms of apoptosis [1] [23], but this phenomenon has never been documented during viral infection. In a preliminary experiment, we first verified that apoptosis induced by cycloheximide and TNF-α was associated with HMGB1 retention on chromatin in HEC-1 cells (Figure 3A).

In uninfected cells, HMGB1 was equally distributed between the nucleus and the cytoplasm (Figure 3B), in keeping with its constant shuttling between these compartments [24]. In the presence of HSV-2, HMGB1 gradually moved from the cytosol and transiently concentrated in the nucleus, suggesting that it was retained on chromatin early after infection (Figure 3B). To test this assumption, mobile and chromatin-bound HMGB1 fractions were separated after cell permeabilization with NP40 as described in [1]. As shown in Figure 3C, chromatin bound (CB) HMGB1 increased until day 2 pi, then fell slightly, concomitantly with HMGB1 extracellular release (Figure 3D). HMGB1 release was time- and dose-dependent, peaked on day 5 pi, and ran parallel to HSV-2-induced necrosis (Figure 2D). For the highest MOI, the leakage of HMGB1 out of the cells starts between day 2 and day 3 pi, which explains why HMGB1 concentration decreases in both nuclear and cytosolic fractions at these time points (Figure 3B). Contrary to previous observations made in post-apoptotic cells [25], extracellular HMGB1 was not nucleosome-associated, as it never copurified with histone in the culture medium (data not shown).

Thus, HSV-2 multiplication and virion release were associated with apoptosis and HMGB1 retention on chromatin, followed by necrosis and extracellular HMGB1 release.

### HMGB1 released during HSV-2 infection induces cell migration and reactivates latent HIV-1

HMGB1 released by HSV-2 infected cells might contribute to signaling of the infection and modify the environment of infected cells *in vivo*. However, HMGB1 extracellular activities can be modulated positively or negatively, by oxidation in necrotic and apoptotic cells [3] [5] [4] and/or by interactions with a large set of molecules of cellular or viral origin [8].

**Figure 4:**
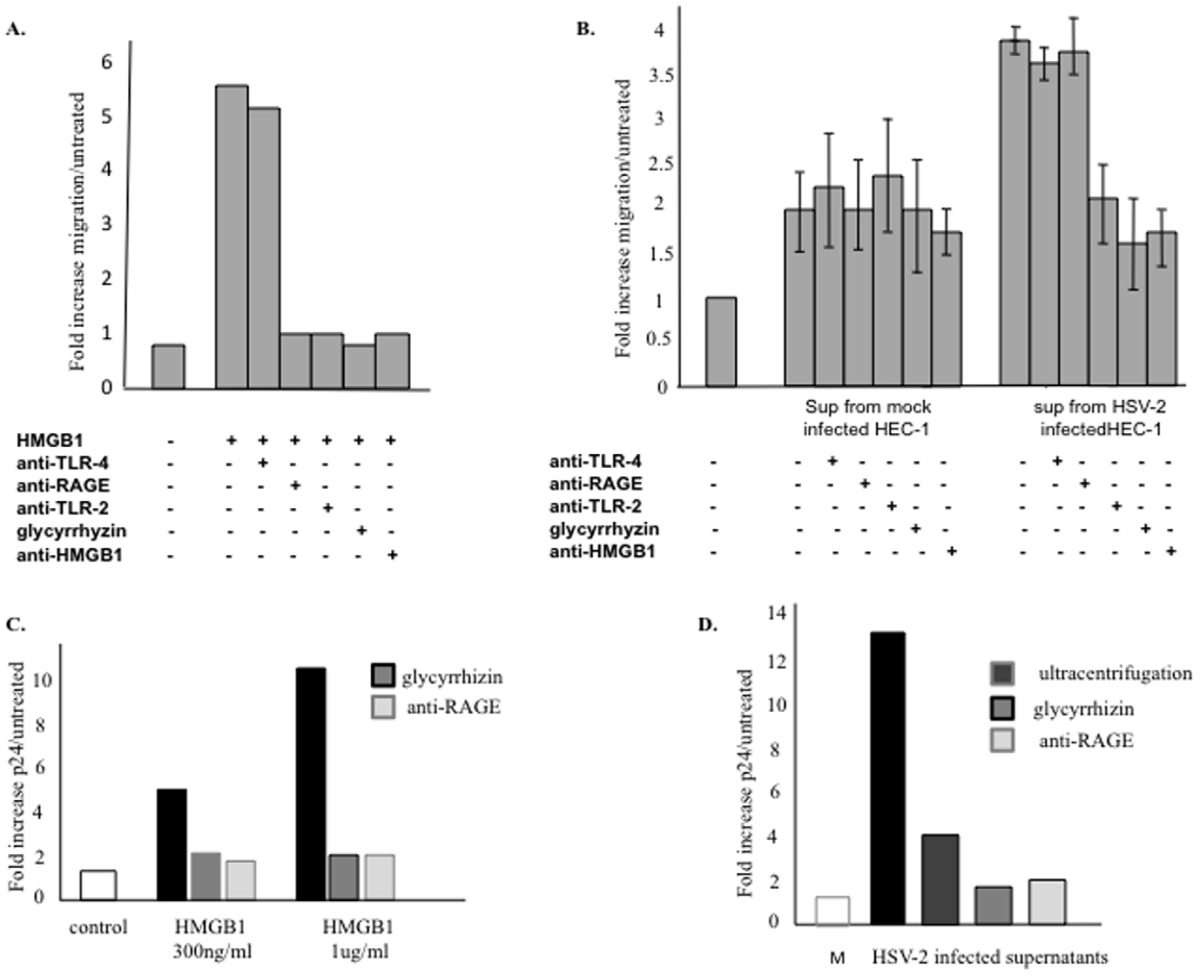
HMGB1 released by HSV-2-infected cells activates fibroblast migration and stimulates HIV-1 expression. MRC5 fibroblasts were subjected to migration assays in the presence of (A) human recombinant HMGB1 or (B) supernatants from mock (M) or HSV-2-infected HEC-1 cells. HMGB1 concentrations reached 270 ng/ml in the supernatants collected from infected cells, versus 50 ng/ml in mock-infected cell media. Control experiments used glycyrrhizin or neutralizing antibodies against HMGB1 or its receptors RAGE, TLR-4 and TLR-2. Data are expressed as -fold increases in cell migration compared to non treated control cells. ACH-2 cells, that contain a latent HIV-1 provirus, were grown in the presence of (C) human recombinant HMGB1 or (D) supernatants from mock (M) or HSV-2-infected HEC-1 cells. P24 antigen was measured by ELISA after 36 h. Data are expressed as the -fold increase in p24 antigen compared with non treated control cells. Virion-free supernatants were obtained by ultracentrifugation. Results are either representative of several experiments (A, C, D) or expressed as the mean and SD of 3 independent experiments (B).

We first examined the chemotactic activity of culture supernatants by using human MRC5 fibroblasts. Preliminary experiments indicated that recombinant human HMGB1 stimulated MRC5 fibroblast migration (Figure 4A) through RAGE and TLR-2. A slight HMGB1-independent increase in cell migration was observed with supernatants from mock-infected cells (Figure 4B), while supernatants of HSV-2-infected cells exhibited strong chemoattractant activity (Figure 4B). This activity was largely mediated by HMGB1 through TLR-2, as it was abrogated by glycyrrhizin, an HMGB1 inhibitor [26], and also by neutralizing anti-HMGB1 and anti-TLR2 antibodies, but not by antibodies blocking RAGE or TLR-4. Similar results were obtained with 3T3 murine fibroblasts (not shown).

We have previously shown that cellular and recombinant forms of human HMGB1 purified from *Escherichia coli* reactivate HIV-1 from post-integration latency in a RAGE-dependent manner, notably in ACH-2 lymphocytes [27] (Figure 4C). As HIV-1 and HSV-2 may functionally interact in the genital mucosa [28],we wondered whether HMGB1 released by HSV2-infected cells might modulate HIV-1 reactivation as well. Thus, ACH-2 cells were exposed to supernatants collected from mock- and HSV-2-infected cells. A 12-fold increase in p24 antigen expression was observed in ACH-2 cells treated with crude supernatants from HSV-2-infected cells relative to the control (Figure 4D). As HSV-2 itself can reactivate latent HIV-1 [29], infectious particles were removed from the supernatants by ultracentrifugation. As expected, HIV reactivation was lowered in the presence of virion-free supernatants but remained still significant compared to the control (Figure 4D). The addition of glycyrrhizin or blocking anti-RAGE antibody totally abrogated HIV-1 reactivation, suggesting that HMGB1 released by HSV-2-infected HEC-1 cells may still play a role in HIV-1 induction in the context of crude cell extracts. Altogether, these results confirm that HSV-2 infection promotes the release of HMGB1 in a biologically active form.

### Detection of HMGB1 in cervico-vaginal secretions from HSV-2-infected women

As HSV-2 infection induced the release of biologically active HMGB1 in vitro, we examined whether active genital HSV-2 infection was associated with HMGB1 release in vivo, by quantifying HMGB1 in cervico-vaginal samples collected from 18 HSV-2-infected women, during HSV-2 recurrence or not. Two techniques were used i.e. a commercial ELISA and an original gel-shift assay that was developed in our laboratory [30]. This assay relies on the ability of HMGB1 to form highly specific and stable complexes with hemicatenated DNA [31]. The correlation between the two methods was excellent (p = 0.0007, Spearman's Rank test), even if some discrepancies were sometimes observed, especially for the highest concentrations of HMGB1 (Table 1).

**Table 1:** Correlations between HMGB1 concentrations and HSV-2 shedding in cervico-vaginal samples collected from HSV-2-infected women.

patient	viral culture	HSV-2 DNA (copies/ml)	HMGB1 (gel shift, ng/ml)	HMGB1 (ELISA, ng/ml)
1	neg	0	0	4.5
2	HSV2	465.5	0	2.8
3	neg	0	3	2.7
4	CMV	0	2	12.1
5	neg	0	10	10.7
6	neg	0	0	5.4
7	HSV2	99500	540	129.3
8	neg	0	5	1.5
9	HSV2	3520	15	21.6
10	neg	0	10	2.6
11	neg	0	0	2.3
12	neg	0	0	2.3
13	neg	0	0	4.8
14	neg	0	23	19.4
15	HSV2	1355000	22	40.4
16	neg	0	550	113.1
17	neg	0	0	2.5
18	HSV2	5250	32	12.5

Eighteen genital samples were collected from women seropositive for HSV-2. Viral culture was performed on Vero cells, and HSV-2 DNA was quantified with real-time PCR. HMGB1 was quantified with a commercial ELISA and a band-shift assay, as described in [32].

**Figure 5:**
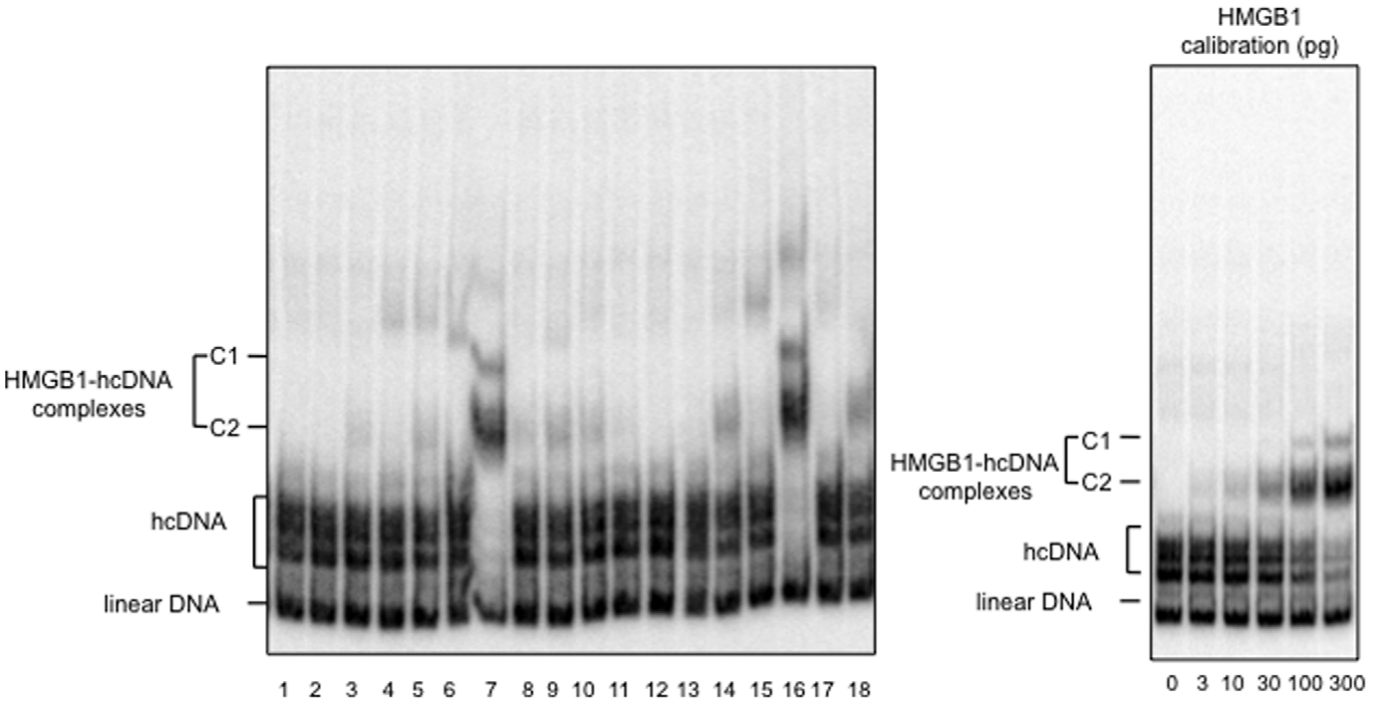
Band-shift assay for HMGB1 detection in cervicovaginal secretions from HSV-2-infected women. Ten microliters of cervicovaginal specimens (#1–18) collected from 18 women seropositive for HSV-2 were mixed with radiolabeled hemicatenated DNA (hcDNA). HMGB1-hcDNA shifted complexes were analyzed by electrophoresis on nondenaturing polyacrylamide gel, using a band-shift assay, as described in (30). The amount of HMGB1 was calculated from the percentage of shifted hcDNA, quantified with ImageJ software (table 1).

Eight out of 18 samples exhibited HMGB1 concentration above 10 ng/ml and two of them contained a very high concentration of HMGB1, above 500 ng/ml HMGB1 as estimated by gel shift assay and above 100 ng/ml by ELISA (Figure 5, Table 1). Importantly, a significant positive correlation was observed between the amount of viral DNA, which reflects viral replication, and HMGB1 concentrations, quantified either by gel shift (p = 0.05, rho value  = 0.46, Spearman's rank test) or by ELISA (p = 0.02, rho value  = 0.56, Spearman's rank test). This result demonstrated that HSV-2 active infection is associated with the release of soluble HMGB1 in vivo as well, at least in the context of genital infections.

## Discussion

We show that HMGB1 expression and dynamics are profoundly altered during HSV-2 infection. Whereas HMGB1 constantly shuttles between the nucleus and cytoplasm of HSV-2-uninfected cells, it gradually concentrated in the nucleus and co-purified with chromatin during the pro-apoptotic phase of the infection that occurred from 24 to 48 hours post-infection in HEC-1 cells. This is reminiscent of the HMGB1 retention observed in some models of chemically induced apoptosis [1], even though this observation has been challenged [23] [17] [3] and may depend either on the nature of the apoptotic signal or on the cell type. Our data provide the first evidence that virus-induced apoptosis can also stimulate HMGB1 association with chromatin, a phenomenon that peaked when virus production was already at a plateau. Nevertheless, virus-induced necrosis still occurred later during the infection, promoting massive release of HMGB1. HMGB1 release has previously been reported during *in vitro* infection with RNA viruses such as Dengue virus [32] [33] or HIV-1[34]. However, our study is the first detailed investigation of HMGB1 release and activity in a model of lytic DNA virus, where a balance between pro- and anti-apoptotic signals has been shown to modulate the cell death process and, as a possible consequence, the severity of the disease [20]. Our results are notably different from those obtained with HIV-1-infected lymphocytes, where HMGB1 release has been linked to both apoptosis and necrosis, being inhibited in the presence of apoptosis inhibitors [34]. The process is different during HSV-2 infection, as inhibition of apoptosis by Z-VAD led to increased necrosis, indicating that HSV-2-induced apoptosis delays cell necrosis and subsequent HMGB1 release. This phenomenon would be highly beneficial to the virus, as it could allow the viral cycle to be completed without interference from HMGB1-dependent immune responses. Contrary to the widely accepted notion that apoptosis is a first line of defence against viral infection; virus-induced apoptosis might rather favour virus multiplication *in vivo* by limiting necrosis, a process that leads to inflammation and activation of innate immune responses.

It has been reported that HMGB1 released during apoptosis or necrosis is largely oxidized [35], which may affect its stimulatory activity [3] [5]. However, we show here that late release of HMGB1 by infected epithelial cells displays at least two biological activities that are highly relevant to herpetic infection. First, culture medium from HSV-2-infected cells exhibited strong chemoattractant activity for fibroblasts, mostly owing to the presence of HMGB1. This finding is important, given the well-known role of fibroblasts in herpetic lesions [36] and wound healing [37], and suggests that HMGB1 may contribute to the healing of herpetic genital lesions. In our model, fibroblast migration was dependent on HMGB1-TLR2 interaction but did not require RAGE, as previously observed in other cell models [38].

Reversely, the alarmin activity of HMGB1 may be detrimental, notably in HSV-2/HIV co-infection. The known deleterious impact of HSV2 infection on HIV disease progression has been at least in part linked to direct interplay between the two viruses [10]. The strong increase in HIV-1 expression in chronically infected T cells exposed to HSV-2-infected HEC-1 cell supernatants, and its partial reversal in the absence of viral particles, confirms the major impact of HSV-2 particles on retrovirus expression [29]. However, we also found that HMGB1 is the major soluble factor released by HSV-2 infected cells that contributes to HIV-1 activation, since its neutralization by glycyrrhizin totally abrogated HIV-1 reactivation by infectious virus-free supernatants. We and others have previously shown that HMGB1 can reactivate HIV-1 both *in vitro* and *ex vivo* [27], [39]. This is therefore confirmed here, as HMGB1 released in response to HSV-2 infection specifically activated - though moderately- HIV-1 in latently infected cells, in a RAGE-dependent manner.

The endometrial epithelial cells used in our experiments may not be fully representative of lower genital epithelia. However, the relevance of our experimental data is strengthened by the detection of biologically relevant concentrations of HMGB1 in cervico-vaginal secretions from some HSV-2-infected women. HMGB1 has been detected in the blood of patients with various conditions, but few data are available on specimen collected from sites of local inflammation or cell damage. Our study is the first to show the presence of significant amounts of HMGB1 in the female genital tract. Since cervico-vaginal washing fluids are rather complex media, we quantified HMGB1 by two different methods, a commercial ELISA and a band shift assay that is less prone to interfering with masking proteins that might associate with HMGB1 in vivo [30]. Importantly and whatever the method used to quantify HMGB1, a positive correlation was observed between HSV2 replication and HMGB1 concentrations in these samples, which supports the involvement of HSV-2 in HMGB1 release through virus-mediated cell necrosis.

Besides being the first target of sexually transmitted HSV-2, the genital epithelium plays a key role in triggering innate immunity to microbial pathogens [40]. Among other pattern recognition receptors, TLR-2 and TLR-9 have been implicated in anti-HSV-2 immune responses through the recognition of still unknown viral components [41] [40] [42] [16]. The key role of TLR-2 during HSV-2 genital infection has been notably highlighted by a decrease in HSV-2-specific responses observed in TLR2 knock-down mice, intra-vaginally challenged with a viral peptide epitope extended by a TLR-2 agonist [43]. HMGB1 likely contributes to this process by interacting with TLRs, alone or together with nucleosomes [25]. Importantly, we detected no free or HMGB1-associated histones in the supernatants of HSV-2-infected HEC-1 cells, suggesting that HMGB1 was not bound to post-apoptotic nucleosomes (not shown). Alternatively, HMGB1 can form active complexes with CpG DNA, which may enhance [17], [44], [45] or diminish [46] TLR 9-dependent responses. As CpG motifs are abundant in the HSV-2 genome [47], HMGB1 accumulation at the site of HSV-2 infection could contribute to modulating an appropriate innate response, notably through dendritic cell activation, and to coordinating tissue repair in a mutually non exclusive manner. These important issues are currently under investigation in our laboratory.

## Materials and Methods

### Cell lines and virus

HEC-1 cells were from INSERM U743 (Paris, France). ACH2 cells, a subclone of HIV-1-infected human T cells, were from the AIDS Research and Reference Reagent Program (Bethesda, USA). Mouse 3T3 fibroblasts were kindly provided from Institut de Génétique et Microbiologie (Orsay, France) and human MRC5 fibroblasts from R/D Biotech (Besançon, France). Cells were cultured at 37°C with 5% CO_2_ in RPMI medium (ACH-2 and HEC-1 cells) or DMEM (MRC5 and 3T3 cells), supplemented with 10% foetal bovine serum and 1% penicillin/streptomycin (Invitrogen, Cergy-Pontoise, France).

To prepare an HSV-2 stock, subconfluent MRC5 fibroblasts were inoculated with a clinical HSV-2 isolate of genital origin, at a multiplicity of infection (MOI) of 0.001 per cell. On day 5 post-infection, the cell supernatant was collected and clarified by centrifugation. The virus stock was subsequently concentrated and purified by ultracentrifugation (228 000 *g*, 2 h, 4°C). The resulting virus pellet was resuspended in RPMI medium. The virus titer was determined as described elsewhere [48]. A single stock (4.2×10^7^ pfu/ml) was stored at −80°C and used for all experiments.

### HMGB1 transcription during HSV-2 infection

HMGB1 mRNAs were quantified in HEC-1 cells with a real-time RT-PCR method, at various times post-infection. Total RNA was extracted using the Trizol method (Invitrogen, Cergy-Pontoise, France). One microgram of RNA was reverse transcribed using 200 U of Moloney murine leukemia virus reverse transcriptase (Invitrogen, Cergy-Pontoise, France).

PCR reactions were performed in a final volume of 20 µl, containing master SYBRGreen (Roche Diagnostics, Meylan, France) and 300 nM each HMGB1 primer, as previously described [49].

Delta CT values were defined as the absolute difference between the cycle threshold (CT) of HMGB1 RNA and the house-keeping gene encoding 18S rRNA. Gene expression data were analyzed with the 2^ - ΔΔCT^ method [50].

### Cell viability analysis

Cell viability was assessed with the trypan blue exclusion method. The role of apoptosis and necrosis in cell death was examined with specific methods, as follows.

### Lactate dehydrogenase (LDH) assay

Cell necrosis was evaluated by quantifying the release of the stable cytosolic enzyme lactic dehydrogenase (LDH), using the Cytotoxicity Detection kit from Roche Diagnostics (Meylan, France) as recommended by the manufacturer. In one control well, NP40 was used at a final concentration of 0.1% to determine maximal LDH release.

The percentage of necrosis, evaluated by the ratio between the amount of released LDH in the medium over the maximal LDH release, was defined as OD _experimental value_ /OD _control_ *100.

#### Caspase 3 and PARP cleavage analysis

At appropriate times post-infection, mock and HSV-2-infected cells were lysed in RIPA buffer (0.25% sodium deoxycholate, 0.1% SDS, 1% NP-40 in PBS) containing protease inhibitors. Total protein (5 and 30 µg) was separated by SDS-PAGE and submitted to western blotting against pro-caspase 3 and PARP proteins, respectively. In some experiments, cells were treated with the caspase inhibitor Z-VAD-FMK (Promega, Yerres, France), at a concentration of 40 µM.

### Intracellular HMGB1 dynamics

#### Subcellular distribution of HMGB1

The Subcellular Proteome Extraction kit (Calbiochem) was used to separate cytosolic, membrane, nuclear and cytoskeleton fractions. HMGB1 was detected in each fraction by western blotting.

#### Cell permeabilisation assay

Chromatin-bound HMGB1 was collected from mock and HSV-2-infected cells after NP-40-induced membrane permeabilization [1] and analyzed by western blotting.

#### HMGB1 release

HMGB-1 released from mock and HSV-2-infected cells was quantified with a commercial ELISA method (Shino test, Kanagawa, Japan).

### Western blotting

Proteins were separated by SDS-PAGE and transferred to nitrocellulose membranes (Hybond, Amersham, Saclay, France). The membranes were blocked for 1 h, then washed and incubated with the following primary antibodies: anti-HMGB1 (clone 4C9), anti-PARP (clone H-250), anti-histone H1 (clone AE-4), anti-pro-caspase3 (clone E-8) and anti-GAPDH (clone 10B8) (Santa Cruz Biotechnology, CA, USA) at 1∶2000 dilution; and anti-LDH (clone EP1566Y) (Abcam Cambridge, UK) at 1∶20 000 dilution.

The secondary antibodies were a 1∶5000 dilution of anti-rabbit immunoglobulin-horseradish peroxidase or anti-mouse immunoglobulin-horseradish peroxidase conjugates (Amersham, Saclay, France). Immune complexes were detected with ECL detection reagents (Amersham, Saclay, France).

### Biological activities of HMGB1-containing supernatants

Supernatants collected on day 5 from mock and infected HEC-1 cell cultures, were either used crude or after removal of infectious particles by ultracentrifugation. The efficiency of this procedure was checked by plaque assay as described [48]. When indicated, the supernatants were treated with the HMGB1 inhibitor glycyrrhizin (250 µM) or 10 µg/ml neutralizing antibodies against RAGE (clone 176902, R&D systems, France), TLR-2 (clone TL2-1, eBioscience) or TLR-4 (clone HTA 125, eBioscience). Protein A-purified neutralizing rabbit anti-HMGB1 IgG (50 µg/ml) was produced and also used for neutralization assays.

### Chemotaxis assay

Chemotaxis assays were performed as previously described [51], with the following minor modifications: mouse 3T3 and human MRC5 fibroblasts and uncoated PVP free-polycarbonate filters (Corning) were used, and cell migration lasted 4 h. Cells remaining on the upper surface of the filters were mechanically removed, and migrating cells were quantified after crystal violet coloration, as described elsewhere [52].

### HIV-1 p24 antigen production by chronically infected lymphocytes

ACH-2 cells were grown in the presence of mock or HSV-2-infected HEC-1 culture supernatants. HIV production was evaluated by measuring p24 antigen with an ELISA method (Innogenetics, Les Ulis, France).

### Detection of HMGB1 in cervicovaginal secretions from HSV-2-infected women

We used a collection of cervicovaginal secretions collected during a randomized study of the impact of anti-herpetic therapy on genital shedding of HIV-1 (ANRS 1212). This study was approved by the research ethics committees of the Ministries of Health of Ghana and the Central African Republic and the London School of Hygiene & Tropical Medicine, and written informed consent was obtained from all the participants. After approval from the scientific committee of this ANRS study, eighteen specimens collected from a group of women, infected by HSV-2 but sero-negative for HIV-1, were analyzed. Cervicovaginal secretions were collected as described elsewhere [13], [53] After a centrifugation step to remove genital cells, supernatants were stored at −20°C until use. Vero cells were used for viral culture, and HSV-2 DNA was quantified by real time PCR, as described [54].

HMGB1 was quantified in the same samples by using two methods: a commercial ELISA test (Shino test, Kanagawa, Japan) and a sensitive in-house gel shift assay, that was performed as previously described [30]. Briefly, 10 µl of cervicovaginal specimens were mixed with radiolabeled hemicatenated DNA (hcDNA) and HMGB1-hcDNA shifted complexes were analyzed by electrophoresis on nondenaturing polyacrylamide gel. The amount of HMGB1 was calculated from the percentage of shifted hcDNA, quantified with ImageJ software.

Correlations between the two methods used to quantify HMGB1, and between HMGB1 and HSV-2 DNA concentrations, were identified with the Spearman*'*s rank correlation test (software Stata, version, 10.0).
